# Concomitant occurrence of FXTAS and clinically defined sporadic inclusion body myositis: report of two cases

**DOI:** 10.3325/cmj.2017.58.310

**Published:** 2017-08

**Authors:** Mirna Lechpammer, Verónica Martínez Cerdeńo, Michael Ryan Hunsaker, Mina Hah, Hilary Gonzales, Steve Tisch, Ronald Joffe, Roger Pamphlett, Flora Tassone, Paul J. Hagerman, Samuel J. Bolitho, Randi J. Hagerman

**Affiliations:** 1 Department of Pathology and Laboratory Medicine, University of California Davis, Medical School, Sacramento, CA, USA; 2 MIND Institute, University of California Davis, Sacramento, CA, USA; 3 Institute for Pediatric Regenerative Medicine and Shriners Hospital for Children of Northern California, Sacramento, CA, USA; 4 Center for Integrative Neuroscience and Human Behavior, Department of Psychology, University of Utah, Salt Lake City, UT, USA; 5 Department of Psychiatry and Behavioral Sciences, University of California Davis, Medical School, Sacramento, CA, USA; 6 Department of Biochemistry and Molecular Medicine, University of California Davis, Medical School, Sacramento, CA, USA; 7 Department of Pathology, University of Sydney, School of Medical Sciences, Sydney, New South Wales, Australia; 8 Department of Neurology, St Vincent’s Hospital, Darlinghurst, New South Wales, Australia; 9 Department of Pediatrics, University of California Davis, Medical School, Sacramento, CA, USA

## Abstract

This report describes unique presentations of inclusion body myositis (IBM) in two unrelated patients, one male and one female, with genetically and histologically confirmed fragile X-associated tremor/ataxia syndrome (FXTAS). We summarize overlapping symptoms between two disorders, clinical course, and histopathological analyses of the two patients with FXTAS and sporadic IBM, clinically defined per diagnostic criteria of the European Neuromuscular Centre. In case 1, a post-mortem analysis of available brain and muscle tissues is also described. Histopathological features (rimmed vacuoles) consistent with clinically defined IBM were detected in both presented cases. Postmortem testing in case 1 revealed the presence of an FMR1 premutation allele of 60 CGG repeats in both brain and skeletal muscle samples. Case 2 was a premutation carrier with 71 CGG repeats who had a son with FXS. Given that FXTAS is associated with immune-mediated disorders among premutation carriers, it is likely that the pathogeneses of IBM and FXTAS are linked. This is, to our knowledge, the first report of these two conditions presenting together, which expands our understanding of clinical symptoms and unusual presentations in patients with FXTAS. Following detection of a premutation allele of the *FMR1* gene, FXTAS patients with severe muscle pain should be assessed for IBM.

Fragile X syndrome (FXS) is a neurodevelopmental disorder caused by expansions of a CGG trinucleotide repeat element within the 5′ non-coding region of the fragile X mental retardation 1 (*FMR1*) gene (1,2). Full mutation alleles (>200 CGG repeats) generally result in a complete deficit of *FMR1* protein (FMRP) (3,4). Fragile X-associated tremor ataxia syndrome (FXTAS) is characterized by white matter changes with generalized brain atrophy and the presence of spherical intranuclear inclusions in neurons and astrocytes (5,6). Clinically, FXTAS presents by the core features of gait ataxia and intention tremor, neuropathy, psychiatric problems, and cognitive impairment that includes executive dysfunction and memory deficits leading to dementia in approximately 50% of males (7,8).

Inclusion body myositis (IBM) is a form of idiopathic inflammatory myopathy, which generally develops in adults over the age of 50 years, with higher prevalence in males. IBM is unresponsive to immunosuppressive therapy, which differentiates it from the other myopathies (9,10). IBM is characterized by insidious clinical onset of slowly progressive, usually asymmetric, proximal and distal muscle weakness. Clinical diagnosis of IBM is obtained by muscle biopsy with determination of clinical laboratory features, electromyography, and magnetic resonance imaging (MRI) of muscles (10). Currently, there is no curative treatment for FXTAS or IBM (11-13).

Although FXTAS and IBM may have similarities in clinical presentations, these two conditions were not previously reported to occur simultaneously in the same patient. Here we describe two cases of genetically and histologically confirmed FXTAS with a concomitant clinical diagnosis of IBM.

## CASE REPORT 1

Patient 1, a Caucasian male, US Air Force WWII veteran pilot and a retired physician, presented at age 75 with a slight foot drop, which progressed to ataxia and falling with no tremor (Table 1). Spinal MRI was normal at that time, as were metabolic, endocrine and heavy metal poisoning peripheral venous blood analyses, suggesting a primary motor neuron disease. At 77 years of age, the patient underwent a total hip replacement under general anesthesia. The patient continued to have progressive lower extremity weakness and he developed upper extremity weakness after his surgery. At 79 years of age, the patient had another total hip replacement, followed a year later by a shoulder replacement. A muscle biopsy was performed, along with electron microscopy, which was inconclusive upon review by muscle pathologists. Upon evaluation by a neurologist and a rheumatologist, the patient was clinically diagnosed with IBM (Clinically Defined IBM per European Neuromuscular Centre diagnostic criteria) (14). He was reported to have serum creatine kinase (CK) levels that reached 1800 U/L (normal <171 U/L). The following year, the patient was suspected to have had a septic embolic stroke after falling and sustaining an open ankle fracture. By this point in time, the patient had become confined to bed because of significantly progressive weakness and ataxia. The patient did not present with tremor. The family reports no intellectual or cognitive decline. He died at the age 88 from respiratory failure.

**Table 1:** Case 1: medical history timeline

Year	Summary of initial and follow-up visits	Diagnostics / Interventions
1999	Primary motor neuron disease; foot drop, ataxia, falling	Spinal magnetic resonance imaging, metabolic, endocrine and heavy metal poisoning blood analyses (negative)
2001	Progressive lower extremity weakness	Total hip replacement (not related to neurological symptoms)
2003	Develops upper extremity weakness	Total hip replacement (not related to neurological symptoms)
2004	Clinical diagnosis of inclusion body myositis	Shoulder replacement (not related to neurological symptoms); muscle biopsy, electron microscopy
2005	Septic embolic stroke following open fracture of the ankle; progressive weakness and ataxia	Supportive care; patient confined to bed
2012	Death due to respiratory failure	
2013	Diagnosis of fragile X-associated tremor/ataxia syndrome	Postmortem analysis: 60 CGG repeats in the brain and skeletal muscles

Postmortem testing revealed the presence of an *FMR1* premutation allele of 60 CGG repeats in both skeletal muscle and brain samples. No blood sample was available to test if constitutive DNA also holds the permutation allele.

**Figure 1:**
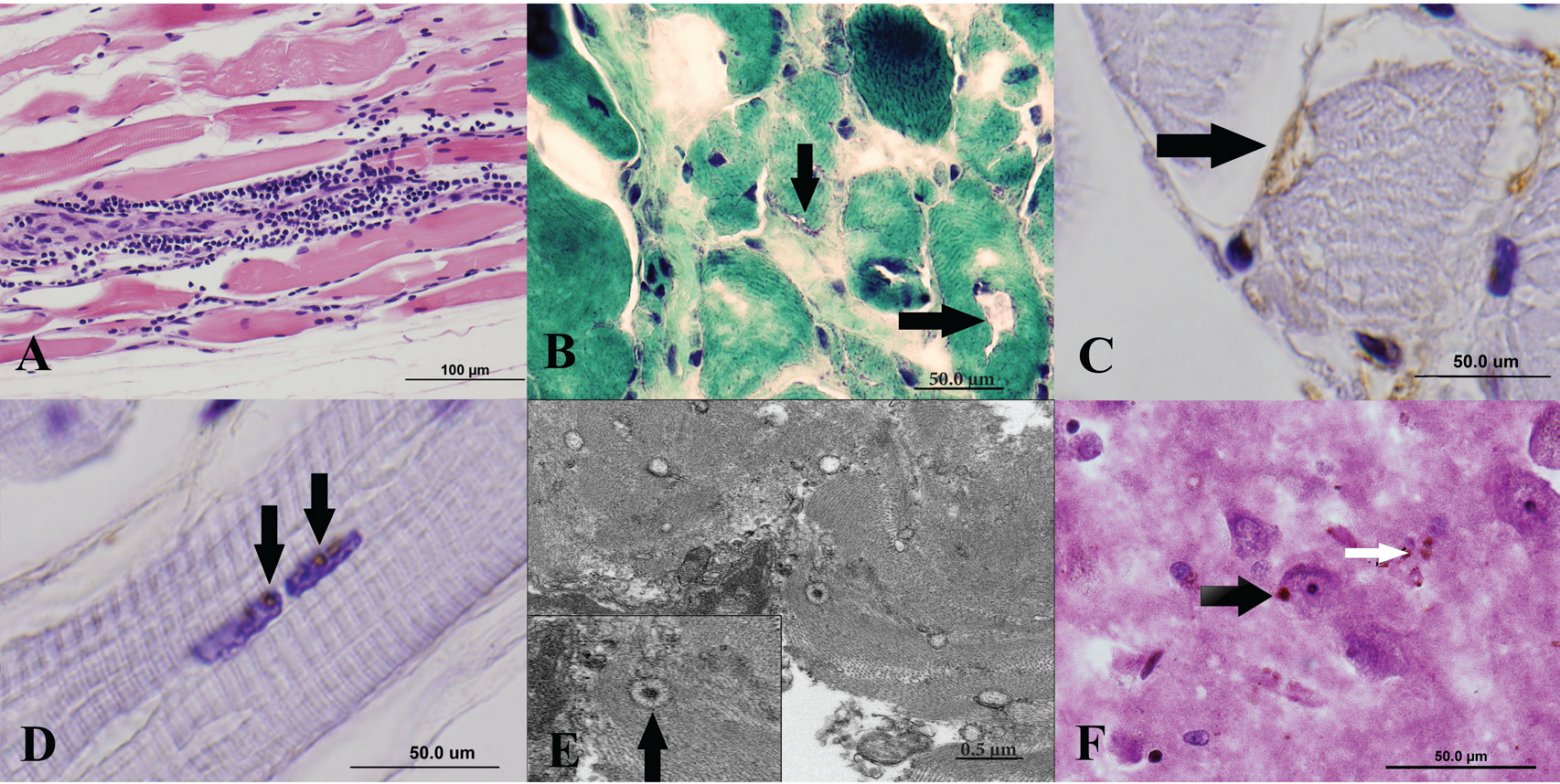
Histopathological and ultrastructural features in the skeletal muscle and brain of Case 1 showing chronic inflammatory myopathy and rimmed vacuoles along with neuronal and glial inclusions. **A.** Chronic inflammatory myopathy, hematoxylin and eosin (H&E) stain-400 × magnification. **B.** Black arrows are pointing to rimmed vacuoles, Trichrome Stain, 600 × oil magnification. **C.** Black arrow is pointing to TAR DNA-binding protein 43 (TDP-43) positive inclusions in cytoplasm of muscle fiber, H&E Stain, 1000 × magnification (oil). **D.** Black arrows are pointing to TDP-43 positive inclusions in muscle nuclei, H&E stain, 1000 × magnification (oil). **E.** Black arrow is pointing to rare muscle inclusions, transmission electron microscopy. **F.** H&E plus ubiquitin, 1000 × magnification (oil) in brain tissue; black arrow points to neuronal inclusion and white arrow points to glial inclusion.

Findings in skeletal muscle included chronic inflammatory myopathy and rare rimmed vacuoles (Figure 1A). Examination of paraffin and frozen hematoxylin and eosin (H&E) sections showed marked variations in fiber size. Some round atrophic and angulated fibers and rare nuclear bags were seen. Internalized nuclei were increased. There was evidence of myophagocytosis, degenerating and regenerating fibers, and moderate chronic inflammatory infiltrate surrounding and infiltrating muscle fibers with scattered necrotic fibers with a predominance of T cells (CD3 positive). Gomori Trichrome showed prominent interstitial fibrosis. Rare rimmed vacuoles were seen on Trichrome stain (Figure 1B). There was no evidence of ragged red fibers. Nicotinamide adenine dinucleotide (NADH) staining has revealed moth-eaten and targetoid fibers. Succinate dehydrogenase stain showed patterns similar to NADH preparation.

Histopathological features (endomysial inflammatory infiltrate and rare rimmed vacuoles) were consistent with diagnosis of IBM in appropriate clinical setting. Aggregates of TAR DNA-binding protein 43 (TDP-43) were observed in the cytoplasm of 5%-7% myofibers (Figure 1C), a finding supportive of IBM. Very rare nuclei contained round to oval TDP-34 positive inclusions, which morphologically resemble those observed in brain specimens (Figure 1D). Significance of such a finding is unclear. Tubulo-filamentous inclusions were not seen on the transmission electron microscopy. There was no evidence of perifascicular atrophy, making diagnosis of dermatomyositis unlikely. Congo red staining was negative.

Electron microscopic studies showed very rare, non-membrane bound, non-filamentous inclusions (Figure 1E) and evidence of degeneration and regeneration of myofibers with moderate disarray of the contractile apparatus. The mitochondria appeared normal in number, internal structure, size, and distribution. There was no evidence of inclusions in the cytoplasm or nucleus of myofibrils or endothelial cells, and the vessels were unremarkable.

Postmortem neuropathological analysis revealed ubiquitin-positive intranuclear inclusions typical of FXTAS present in neurons and astrocytes (Figure 1F) in various regions of the brain including hippocampus, frontal cortex, and cerebellum. The inclusions in each area of the brain were present in different percentages (Table 2). Other brain findings included mild atrophy and evidence of small vessel disease (arteriolosclerosis) in the white matter.

**Table 2:** Number of inclusions in astrocytes and neurons of various parts of patient brain for case 1

Brain area	Inclusions (%)	
	astrocytes	neurons
Hippocampus – dentate gyrus	12.41	2.39
Hippocampus – region CA1	8.78	3.64
Frontal cortex	8.40	5.00
Cerebellum – granule cell layer	7.50	2.23

## CASE REPORT 2

Patient 2, a Caucasian Australian female, was a known premutation carrier with 71 CGG repeats who had a son with FXS. She presented at age 61 with difficulty walking and was found to have a foot drop bilaterally (Table 3). Wasting of the small muscles in both hands was noted with wasting and weakness in the distal lower limbs. Knee and ankle reflexes were absent. No sensory changes or fasciculations were seen. The clinical impression after neurological review was suggestive of anterior horn cell pathology. Nerve conduction studies and electromyography were performed, revealing an atypical mixed picture of myopathic and neurogenic features suspicious of amyotrophic lateral sclerosis. A muscle biopsy revealed many necrotic and regenerating myofibers throughout the sample, but no endomysial chronic inflammatory cells were observed. Atrophic myofibers and scattered hypertrophic myofibers were seen. Focal increases in interfiber connective tissue and lipids were present. Rimmed vacuoles were seen in many myofibers, highlighted with acid phosphatase; however, no ragged-red myofibers were seen. Sarcolemmal immunostaining for major histocompatibility complex 1 was increased in all myofibers. Atrophic myofibers and scattered hypertrophic myofibers were seen. The COX negative myofibers were less than 1% of the total number. Moth-eaten myofibers were commonly seen. Histological diagnosis was consistent with inclusion body myositis, and an unsuccessful trial of steroids (prednisone, 25 mg per day for two weeks) was undertaken. The patient was followed over the course of 5 years, but her symptoms did not change significantly.

**Table 3:** Case 2: medical history timeline

Year	Relevant medical and family history	
1987	Son diagnosed with fragile X syndrome	
1992	Diagnosed as a carrier with 71 CGG repeats	

Year	Summary of initial and follow-up visits	Diagnostics / Interventions
2010	Clinical diagnosis of inclusion body myositis (IBM) Foot drop bilaterally, wasting in distal lower limbs, absent knee and ankle reflexes	Nerve conduction studies, Electromyography and muscle biopsy
2010	No response to therapy	Prednisone (25 mg/day) for 14-day trial
2010 – 2015	No significant change in the clinical status	Observation
2015	Diagnosis of fragile X-associated tremor/ataxia syndrome Head tremor, deterioration of walking and balance, numbness in the feet	Clinical evidence of sensory neuropathy in the lower limbs Gait ataxia not consistent with IBM

Five years after the diagnosis of IBM, a head tremor emerged. Walking and balance began to deteriorate more rapidly, with the patient noting numbness in the feet. Clinically, there was evidence of sensory neuropathy in the lower limbs. In addition to a bilateral high-stepping gait, there was gait ataxia out of proportion to the chronic weakness associated with the pre-existing myopathy. She was subsequently diagnosed with FXTAS.

## DISCUSSION

The two case reports presented here on the concomitant occurrence of FXTAS and sporadic IBM expand the awareness of medical community on the complexity of clinical presentations of these two, seemingly pathogenically unrelated neurological disorders. Although the two patients were not managed by the same multidisciplinary team of health care professionals – case 1 was diagnosed in the USA and case 2 in Australia – the two countries abide by congruent medical practices and standards, which adds to the credibility of this joint report.

For case 1, it was not known at the time of the onset of patient’s illness that his granddaughter had the premutation and learning problems. Following the diagnosis of the granddaughter, doctors did not consider the carrier status of the grandfather or reconsider his diagnosis until after death, when neuropathology studies demonstrated FXTAS, and appropriate genetic counseling was provided to the family. The foot drop as a first symptom is not seen in FXTAS, but the subsequent falling and ataxia are typical for FXTAS. The severity of the muscle weakness goes beyond what is usually seen in FXTAS, and case 1 never developed tremor, so the focus was on his muscle workup leading to the IBM diagnosis. However, muscle pain and weakness are common features of FXTAS (7,8). The presence of IBM is likely to increase the chance that FXTAS will develop in an aging carrier, as seen in case 2, reflecting the earlier observation in a cohort study of female carriers with the premutation and immune-mediated disorders (IMD) (15,16).

It is not clear how the RNA-mediated events – collectively termed “RNA toxicity” (17-20) – rooted in increased expression levels of expanded FMR1 mRNA, as observed in permutation carriers, predispose to IMDs. This predisposition could possibly involve either or both direct effects – protein sequestration of splicing factors interfering with their functions as regulator of alternative splicing and of the DROSHA/DGCR8 complex (a key player in miRNA biogenesis), or effects of mitochondrial dysfunction (16,21). The dysregulation of miRNAs is thought to predispose to IMD particularly in women with the premutation (16). In a study of 344 women with the premutation, 45% had at least one IMD involving multiple organs, a significantly higher rate than controls without the premutation. The most common problem was immune-mediated thyroid disorder in 24%, with fibromyalgia in 10%, and less common disorders, including rheumatoid arthritis in 4%, Sjögren’s syndrome in 2.6%, systemic lupus erythematosus in 2.0%, and multiple sclerosis in 2.0% (16). Although case 2 did not have a history of an immune-related disorder, she otherwise did epidemiologically fit into the observation on the increased risk of developing IMD in aging female premutation carriers. For women with FXTAS older than 40 years of age, the proportion with IMD is estimated to be as high as 73% (16). The odds ratio for developing an IMD in women with FXTAS is 2.6% (95% CI 1.2 - 5.6, *P* = 0.015) compared with women carriers without FXTAS (16). IMDs are less common in males with the premutation, although case 1 is an example of an IMD in an older man with FXTAS, and it is likely that both disorders began simultaneously. IMDs can also develop in those with the premutation because the cytokine levels are lower in carriers as they age, making them more susceptible to both infections and IMDs (22). Because muscle pain, often leading to a diagnosis of fibromyalgia, is common, particularly in women with FXTAS (4,16,23), perhaps assessment of creatine kinase levels is warranted as a screen for IBM, which may be more common than currently considered in those with FXTAS.

The histopathology of IBM shows endomysial chronic inflammation, invasion of non-necrotic muscle fibers by inflammatory cells, variable number of rimmed vacuoles, and 15-18-nm tubulofilaments on electron microscopy. Perifascicular atrophy is absent. Amyloid deposition in vacuolated muscle fibers can be demonstrated by Congo red staining. CD8-positive T cells are predominant and invade the non-necrotic fibers (9,10). Described histological findings may not all be present in patients with a clinical IBM (10,14).

Mitochondrial abnormalities are another pathological feature of IBM. These consist mainly of ragged red fibers and reduced cyclooxygenase activity (10). In the presented cases, mitochondrial disease was ruled out by electron microscopy and cyclooxygenase and succinate dehydrogenase activities. Further studies detecting mutations for mitochondrial disease were not performed.

In summary, we described a review of symptoms and histological analysis of two unrelated patients with FXTAS and concomitant, sporadic, clinically defined IBM. This is, to our knowledge, the first report of these two conditions presenting together, which expands our understanding of clinical symptoms and unusual presentations in patients with FXTAS.

FXTAS is reported as early as 30 years of age (24). However, it is known to occur later in life together with other disorders, including Alzheimer disease, Parkinsonism, and Prader-Willi phenotype (3,7,25). IMDs are also often co-morbid with FXTAS (11,16,22). Presented cases emphasize the need to consider FXTAS with the onset of any neurological symptoms in aging premutation carriers, even when the presentation is atypical. Documenting the presence of FXTAS inclusions clarifies the diagnosis of definite FXTAS, as laid out within the diagnostic criteria of this disorder (5,17).
